# The mosaic architecture of *Aeromonas salmonicida* subsp. *salmonicida* pAsa4 plasmid and its consequences on antibiotic resistance

**DOI:** 10.7717/peerj.2595

**Published:** 2016-10-27

**Authors:** Katherine H. Tanaka, Antony T. Vincent, Mélanie V. Trudel, Valérie E. Paquet, Michel Frenette, Steve J. Charette

**Affiliations:** 1 Institut de biologie intégrative et des systèmes, Québec, Canada; 2 Institut universitaire de cardiologie et de pneumologie de Québec, Québec, Canada; 3 Département de biochimie, de microbiologie et de bio-informatique, Université Laval, Québec, Canada; 4 Groupe de recherche en écologie buccale (GREB), Université Laval, Québec, Canada

**Keywords:** *Aeromonas salmonicida* subsp. *salmonicida*, Plasmid, pAsa4, Insertion sequence, Integron, Comparative genomics, Chloramphenicol, Sulfonamide, Tetracycline, Antibiotic resistance

## Abstract

*Aeromonas salmonicida* subsp. *salmonicida*, the causative agent of furunculosis in salmonids, is an issue especially because many isolates of this bacterium display antibiotic resistances, which limit treatments against the disease. Recent results suggested the possible existence of alternative forms of pAsa4, a large plasmid found in *A. salmonicida* subsp. *salmonicida* and bearing multiple antibiotic resistance genes. The present study reveals the existence of two newly detected pAsa4 variants, pAsa4b and pAsa4c. We present the extensive characterization of the genomic architecture, the mobile genetic elements and the antimicrobial resistance genes of these plasmids in addition to the reference pAsa4 from the strain A449. The analysis showed differences between the three architectures with consequences on the content of resistance genes. The genomic plasticity of the three pAsa4 variants could be partially explained by the action of mobile genetic elements like insertion sequences. Eight additional isolates from Canada and Europe that bore similar antibiotic resistance patterns as pAsa4-bearing strains were genotyped and specific pAsa4 variants could be attributed to phenotypic profiles. pAsa4 and pAsa4c were found in Europe, while pAsa4b was found in Canada. In accordance with their content in conjugative transfer genes, only pAsa4b and pAsa4c can be transferred by conjugation in *Escherichia coli*. The plasticity of pAsa4 variants related to the acquisition of antibiotic resistance indicates that these plasmids may pose a threat in terms of the dissemination of antimicrobial-resistant *A. salmonicida* subsp. *salmonicida* bacteria.

## Introduction

The ubiquitous waterborne Gram-negative bacterium *Aeromonas salmonicida* subsp. *salmonicida* is the causative agent of furunculosis, a disease that affects aquaculture operations worldwide (Derome et al., 2016). The main treatments for this disease are vaccination and antibiotics. Vaccination was shown to be efficient but is expensive and may cause major side effects (Dallaire-Dufresne et al., 2014). Antibiotics are becoming increasingly less effective against *A. salmonicida* subsp. *salmonicida* due to the spread of antibiotic resistance genes. For example, more and more antibiotic-resistant *A. salmonicida* subsp. *salmonicida* strains are being isolated and characterized, many of them bearing resistance genes on plasmids (McIntosh et al., 2008; Piotrowska & Popowska, 2015; Sorum et al., 2003; Vincent et al., 2016a; Vincent et al., 2014b).

In *A. salmonicida* subsp. *salmonicida*, insertion sequences (ISs) are responsible for several genomic modifications (Vincent et al., 2016b). ISs are made of a transposase gene and inverted repeats. Some ISs are involved in virulence loss when *A. salmonicida* subsp. *salmonicida* is under stressful conditions (IS*AS1*, IS*AS2* and IS*AS11*) (Gustafson, Chu & Trust, 1994; Tanaka et al., 2012). Furthermore, many plasmid variants display transpositions or IS-mediated recombinations when compared to their reference (IS*AS*5 in many plasmids, IS*Ec*9 in pSN254b) (Attéré et al., 2015; Najimi et al., 2009; Trudel et al., 2013; Vincent et al., 2014b). Given the high number of ISs in the genome of this bacterium (Studer, Frey & Vanden Bergh, 2013; Vincent et al., 2016b), we hypothesize that ISs play a role in plasmid reshaping (Tanaka, Frenette & Charette, 2013).

The large plasmid pAsa4 from *A. salmonicida* subsp. *salmonicida* carries genes that provide resistance against chloramphenicol, spectinomycin, streptomycin, sulfonamides, tetracycline, mercury, and quaternary ammonium compounds (Reith et al., 2008). Except for tetracycline resistance, these genes are located in Tn*21*, a non-composite transposon. Tn*21* is a widespread replicative transposon that also carries another mobile element, the integron In*2* (Liebert, Hall & Summers, 1999). The complete sequence of pAsa4 was first described in reference strain A449, which originated from France (Reith et al., 2008). Genotyping done in a previous study has shown that some *A. salmonicida* subsp. *salmonicida* isolates likely bear pAsa4 but do not display the expected antibiotic resistance profile (Vincent et al., 2014b). This suggests that pAsa4 variants may have evolved from a common replicon backbone, but do not share the same antibiotic resistance genes.

We used next-generation sequencing (NGS) on two isolates, one from the province of Quebec (Canada) and one from Switzerland, suspected of carrying pAsa4 variants based on preliminary genotyping and antibiotic resistance profiles, to obtain the complete sequences of the two plasmids. Both plasmids exhibited marked differences from the original pAsa4 plasmid from the reference strain A449 and from each other. A detailed analysis of these pAsa4 variants is presented.

## Material and Methods

### Bacterial isolates, growth conditions, antibiotic resistance profiles, and conjugation assays

The 129 *A. salmonicida* subsp. *salmonicida* strains listed in Table S1 were included in this study. All strains were grown on furunculosis agar (10 g of Bacto-Tryptone, 5 g of yeast extract, 1 g of L-tyrosine, 2.5 g of NaCl, and 15 g of agar per liter of distilled water) or tryptic soy agar (TSA) for two or three days at 18 °C (Hänninen & Hirvelä-Koski, 1997). *Escherichia coli* DH5α was grown on lysogeny broth (LB) agar for one day at 37 °C. Disk diffusion assays using chloramphenicol (30 μg), florfenicol (30 μg), sulfamethoxazole/trimethoprim (SXT) (23.75/1.25 μg), and tetracycline (5 μg) disks (Becton Dickinson, Franklin Lakes, NJ, USA) were performed for strains listed in Table S1 as done previously (Vincent et al., 2014b).

Bacterial conjugation assays have been done as previously described (Boyd et al., 2008). *A. salmonicida* A449, 01-B522 and JF2267 (donor strains) were pre-cultivated in 2 ml of tryptic soy broth (TSB) at 18 °C overnight. *E. coli* DH5α (recipient strain) was pre-cultivated in 2 ml of LB at 37 °C for the same period of time. For each conjugation experiment, cultures of donor and recipient cells (1 ml each) were harvested by centrifugation at 17,200 × *g* for 1 min, suspended in 20 μl TSB, mixed together, and spotted on TSA without selection for 24 h at 18 °C. Afterwards, the culture was suspended in TSB, diluted and plated on TSA with either 5 μg/ml tetracycline (pAsa4 and pAsa4b) or 5 μg/ml chloramphenicol (pAsa4 and pAsa4c). Plates were incubated overnight at 37 °C to select against *A. salmonicida* which is psychrophilic (Vincent et al., 2016b). Large colonies were picked and suspended in TSB with appropriate selection. The presence of pAsa4 variants in transformants was confirmed by PCR using primer pairs *traG*, 2, 3, 9 and 10 (Table S2), *A. salmonicida* absence was confirmed by *tapA*. Conjugation assays were performed twice for every pAsa4 variant.

### DNA extraction and sequencing

The total genomic DNA of two isolates (01-B522 and JF2267) was extracted using DNeasy Blood and Tissue kits (Qiagen, Canada) and was sequenced at the Plateforme d’Analyse Génomique of the Institut de biologie intégrative et des systèmes (IBIS, Université Laval). For JF2267, a 650-bp shotgun library was sequenced using 454 GS-FLX+ technology. Isolate 01-B522 was sequenced as previously described (454 GS-FLX+ technology, mate-pair library with 5 kbp fragment size and 1,500 bp library size) (Vincent et al., 2014a). The reads were assembled *de novo* using Newbler version 2.5.3 with default parameters (Margulies et al., 2005).

### Sequence analysis

Contigs resulting from the assembly of 01-B522 and JF2267 were initially mapped locally on the sequence of the pAsa4 from A449 (GenBank accession number: NC_009349.1) using CONTIGuator version 2.7.4 (Galardini et al., 2011). All contig junctions were manually verified by PCR and Sanger sequencing and links were joined using Consed version 27 (Gordon & Green, 2013).

The assembled plasmids were annotated as follows. Open reading frames (ORFs) were predicted by getorf (available as a part of EMBOSS 6.6.0.0) (Rice, Longden & Bleasby, 2000). All the detected ORFs were then compared to pAsa4 coding sequences using fasta36 (Pearson & Lipman, 1988). Lastly, the remaining ORFs were annotated using Blastn and Blastp (Altschul et al., 1990) against the NCBI non-redundant (nr/nt) database and, if necessary, against the whole genome shotgun database (wgs, Gammaproteobacteria (taxid:1236)). Annotations were manually verified using the Artemis version 16.0.0 visualization tool, and alignments between the assembled pAsa4 were visualized using EasyFig. 2.1 and ACT 13.0.0 (Rutherford et al., 2000; Sullivan, Petty & Beatson, 2011). IS nomenclature follows the one of *A. salmonicida* A449 original Genbank annotation (“IS*AS*” names, which differs from “IS*As*” nomemclature) (Reith et al., 2008). Antibiotic resistance genes were validated with The Comprehensive Antibiotic Resistance Database (CARD) (McArthur et al., 2013). The annotated sequences of pAsa4b and pAsa4c were deposited in GenBank under accession numbers KT033469 and KT033470, respectively.

The average copy number per cell for pAsa4b in 01-B522 and pAsa4c in JF2267 were estimated by mapping the sequencing reads using TAPyR v1.3-beta4 (Fernandes et al., 2011) and by calculating the average coverage using Qualimap 2.0 (Garcia-Alcalde et al., 2012). The copy numbers were standardized against the average coverage of the *gyrB* housekeeping gene (single copy per chromosome).

Contigs from two other *A. salmonicida* strains, RS 534 (NCBI wgs JYFF00000000) (Vincent et al., 2016b) and JF3517 that had been sequenced previously (Attéré et al., 2015) were mapped against pAsa4, pAsa4b, and pAsa4c using CONTIGuator version 2.7.4 (Galardini et al., 2011).

A global alignment of pAsa4b and pAsa4c was performed using *stretcher* (available as a part of EMBOSS 6.6.0.0) (Rice, Longden & Bleasby, 2000), and a custom R script (R Development Core Team, 2015) was used to visualize the number of substitutions by 1,000-bp sliding windows (Data S1) (Zeileis & Grothendieck, 2005). For the heatmap representations, all the ORFs from pAsa4b were compared to the NCBI nucleotide collection (nr/nt) using tBlastn (Altschul et al., 1990). The data was ordered and visualized using a custom R script (Wickham, 2009). *k-means* clustering was used to group target sequence identifiers based on the matrix results in as many clusters that could create reproducible grouping (Data S2) (Hartigan & Wong, 1979).

### PCR analyses

**Figure 1:**
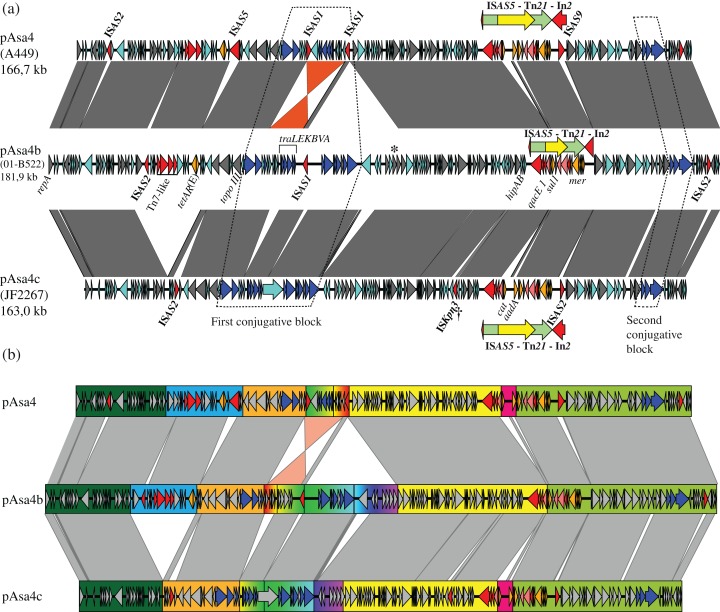
Nucleotide alignment of three plasmid variants: pAsa4, pAsa4b, and pAsa4c. (A) Plasmid alignments and ORF representations were done using EasyFig (Sullivan, Petty & Beatson, 2011). The dark grey bands denote regions of identity. Overall, the identity was more than 94%. The bands of non-contiguous repeat regions were removed for clarity. An inversion between pAsa4 and pAsa4b/c is marked in orange. ORFs are indicated by colored arrows that indicate their deduced function: **Cyan:** hypothetical protein; **Dark grey:** maintenance and replication; **Blue:** type IV secretion system-like conjugative system, contained in two conjugative blocks (dashed lines); **Red:** IS, transposition-associated genes; **Orange:** antimicrobial resistance. The following features have also been annotated: **Asterisk:** phage endonuclease, similar to pAsa4 pseudogene; **Dagger:** IS*1595*-family IS*Kpn3*. A transposon/integron structure (Tn*21*/In*2*) that was common to all pAsa4 plasmids and that is integrated into an IS*AS5* is indicated over each plasmid by nested red, green, and yellow arrows. Besides the transposon, but inside IS*AS5*, an IS*AS9* and an IS*AS2* insertion could be seen for pAsa4 and pAsa4c, respectively. (B) Segments of large insertion/deletion or recombination sequences are highlighted in color. Segments of particular significance are: **Blue:** an insertion/deletion in pAsa4 comprising tetracycline resistance genes *tetAR* (E); **Rainbow progression:** multiple insertions/deletions and an inversion encompassing a conjugative gene region; **Pink:** two events: an IS*CR* insertion comprising the chloramphenicol resistance gene, *cat* and an *aadA1* cassette.

The DNA templates, PCR mixtures, and program cycles were performed as previously described (Trudel et al., 2013), with the exception of the elongation time, which was 1 min per kbp of amplicon. The PCR assays were performed at least twice, and appropriate positive and negative controls were included with each assay. The PCR primers are listed in Table S2. Genotyping primers were designed using PrimerBlast (Ye et al., 2012) at plasmid insertion/deletion sites (junction between segments, Fig. 1B).

## Results

### Complete sequences of the new pAsa4 variants

*A. salmonicida* subsp. *salmonicida* isolate 01-B522 harbored a potential pAsa4 variant based on the genotyping results and antibiotic-resistance profile (Vincent et al., 2014b) (Table 1). Isolate JF2267 displayed genotyping results similar to those of 01-B522, but had a different antibiotic resistance profile (Table 1). To determine the complete sequences of these potential pAsa4 variants, pyrosequencing, PCR and Sanger sequencing were used to assemble the complete plasmid sequences. The reference plasmid pAsa4 is composed of 166,749 bp and 173 ORFs and has a G+C content of 52.8% (Reith et al., 2008) compared to 181,933 bp, 175 ORFs, and a G+C content of 52.48% for 01-B522 pAsa4 variant (pAsa4b), and 163,022 bp, 156 ORFs, and a G+C content of 53.42% for JF2267 variant (pAsa4c). Based on the relative coverage of the sequenced reads compared to *gyrB* coverage, the estimated number of copies of the pAsa4 variants in 01-B522 and JF2267 was 1 in both cases. The contigs alignments of previously sequenced strains RS 534 and JF3517 indicated that they had the same content as pAsa4 and pAsa4c, respectively.

**Table 1:** *A. salmonicida* subsp. *salmonicida* strains bearing pAsa4 variants.

Strain	Source (host)*	Origin*	Antibiotic resistance determined by antibiogram^†^	pAsa4 variant determined by genotyping	Reference
A449	Brown trout	France	TET, CHL	pAsa4	Reith et al. (2008)
RS 534 (A450)	INA	France	TET, CHL	pAsa4	Kay et al. (1981)
01-B522	Brook trout	Quebec (Canada)	SXT, TET	pAsa4b	Daher et al. (2011)
RS 1458	Rainbow trout	Ontario (Canada)	TET	pAsa4b	Attéré et al. (2015)
SHY13-2627	Brook trout	Quebec (Canada)	TET	pAsa4b	Attéré et al. (2015)
SHY13-3799	Brook trout	Quebec (Canada)	TET	pAsa4b	Attéré et al. (2015)
HER1107	INA	INA	TET	pAsa4b	Daher et al. (2011)
JF2267	Arctic char	Switzerland	CHL	pAsa4c	Braun et al. (2002)
JF3517	Turbot	Norway	CHL	pAsa4c	Burr & Frey (2007)
JF3518	Turbot	Norway	CHL	pAsa4c	Burr & Frey (2007)
JF2869	Arctic char	INA	CHL	pAsa4c	Studer, Frey & Vanden Bergh (2013)

**Notes:**

* INA, Information not available or not traceable.

† SXT, sulfamethoxazole/trimethoprim; TET, tetracycline; CHL, chloramphenicol.

pAsa4 and its variants bear many ORFs coding for hypothetical proteins (Fig. 1A, cyan arrows). The plasmids also carry ORFs for their replication and partition and for proteins with other functions (all shown in Fig. 1A as dark grey arrows). Furthermore, two regions bear resistance antibiotic genes (Fig. 1A, orange arrows). Tn*21*, a transposon whose presence was already acknowledged in pAsa4, carries most of the resistance genes via its built-in integron, In*2* (Liebert, Hall & Summers, 1999; Reith et al., 2008). A tetracycline resistance gene and its repressor are located elsewhere on the plasmid (Fig. 1A). Finally, the conjugation-related genes (Fig. 1A, blue arrows) are separated in two loci.

Large insertions or deletions, as well as an inversion, have occurred between the pAsa4 variants, as shown in the sequences alignment (Fig. 1A). These events have mainly occurred between each plasmid’s first conjugative loci, Tn*21*s, and *tetA*(E) flanking sequences. ISs have caused alignment gaps as well. Otherwise, the three pAsa4 variants displayed a high level of sequence identity (from 94 to 99%) for syntenic regions, with pAsa4b being more similar to pAsa4 than pAsa4c. Base substitution count by 1-kbp window between pAsa4b and pAsa4c showed that some regions are more prone to mutations (Fig. S1). In fact, more than 50 substitutions per kilobase occurred upstream from the first transfer genes (Fig. 1B, start of orange segment and Fig. S1 at 45–50 kbp), in a long ORF only predicted in pAsa4c’s first conjugative block (Fig. 1A, longest hypothetical protein in this region and Fig. S1 at 72 kbp) and in a single long ORF found in all plasmids (Fig. S1 at 112 kbp). On the other hand, almost no mismatches were found in the 60-kbp region that comprised Tn*21* and the region downstream from it (Fig. S1, between 125 and 165 kbp).

### Insertion sequences

All pAsa4 variants carry ISs (Fig. 1A, named red arrows). pAsa4b and pAsa4c retained the same IS types that were described in pAsa4, namely IS*AS1*, IS*AS2*, IS*AS5* and IS*AS9* (Reith et al., 2008) (see also GenBank accession number: NC_009349.1). However, no IS shared the same location among all variants, except for the disrupted IS*AS5* nesting the Tn*21* copy. In pAsa4 and pAsa4c, two different ISs (IS*AS9* and IS*AS2*, respectively) are inserted in this disrupted IS (Fig. 1A, downstream of the transposon).

A comparison of transposase sequences using Blast and of inverted repeats using the IS Finder database indicated that there was a member of the IS*1595*-family (Siguier et al., 2006) in pAsa4c (Fig. 1A, dagger). This IS, IS*Kpn3*, has been originally identified in *Klebsiella pneumoniae* plasmid pRDDHA (Verdet et al., 2006). To our knowledge, this was the first identification of this IS in *A. salmonicida*. Based on the Blast search results against the NCBI nr/nt and wgs databases, IS*Kpn3* is present in the *Aeromonas* genus, namely in *Aeromonas media* WS strain (accession number: CP007567.1) and in *Aeromonas dhakensis* SSU strain (accession number: JDWD00000000.1).

### Detailed plasmid architecture

We compared all three pAsa4’s architecture to assess their impact on the plasmid function, including antibiotic resistance (Fig. 1). To facilitate the analysis and the following genotyping, syntenic regions among the variants were grouped together as empirical segments (Fig. 1B, colored rectangles). We investigated the features in each segment as well as their boundaries to infer the causes of these large-scale rearrangements.

A first segment (Fig. 1B, blue rectangle) contained an IS*AS2*, Tn*7*-like transposition protein genes (ABCD), and tetracycline resistance genes (*tetAR*(E)). It was absent from pAsa4c compared to pAsa4 and pAsa4b, which explains why JF2267 was not resistant to tetracycline (Table 1). An imperfect 36-nucleotide inverted repeat flanking this segment in pAsa4 and pAsa4b was not found in pAsa4c at the deletion site, suggesting that it could have been involved in the recombination-deletion process.

**Figure 2:**
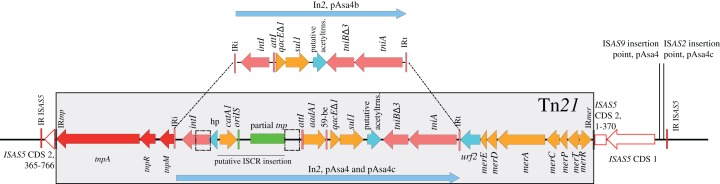
In*2* comparison between pAsa4b and the other variants. Tn*21* differences between the variants are all located in In*2*. Within Tn*21* (shaded rectangle), features are colored using the following: **Red:** Tn*21* transposition genes, **Pink:** In*2* integrase, transposition features and insertion sites, **Cyan:** hypothetical proteins, **Orange:** Antibiotic and mercury resistance genes, **Green:** IS*CR*-related features. Two dotted rectangles represent a repeat region in pAsa4/pAsa4c In*2*, likely caused by *cat*-IS*CR* insertion. This IS is partial, lacking a *terIS* and part of its transposase, suggesting a complex insertion event. The figure also shows where Tn*21* is inserted in IS*AS5*. **Red outline:** IS*AS5* CDS, position called in nucleotide.

Tn*21* and its In*2* spanned over three segments based on this partition (Fig. 1B, yellow, pink and light green rectangles). Two contiguous variations in In*2* are comprised in one segment (Fig. 1B, pink rectangle) that differentiated pAsa4b from pAsa4c and pAsa4, the latter two carrying identical integrons. pAsa4b In*2* bears the integrase, a fused cassette *qacEΔ1 sul1*, a putative acetyltransferase and *tniABΔ3* (Fig. 2). In*2* from pAsa4 and pAsa4c bears an additional *aadA* gene (synonym: *aadA1*) that codes for an aminoglycoside nucleotidyltransferase (ANT(3″)) (Ramirez & Tolmasky, 2010). Also, in pAsa4 and pAsa4c, the *cat* gene (synonym *catA1*, encoding a class A-1 chloramphenicol acetyltransferase) is not inserted as a cassette in In*2*. Instead, it is located between a hypothetical protein ORF and a partial IS*CR* that includes a partial transposase, a 3′ IS*CR* and ori*IS*, but lacks the other components. This insertion is located between partial *intI* duplication. In pAsa4b, neither the insertion nor the duplication was found (Fig. 2).

The regions in the three plasmids harboring most of the conjugative transfer genes also displayed the most differences (Fig. 1B, rainbow colored section). An inversion of the *traLEKBVA* locus and the surrounding region seemed to have occurred in pAsa4. This inversion is flanked by two inverted IS*AS1*s, which are in the appropriate position to have mediated the rearrangement. Only one of the two IS*AS1* was found at this position in pAsa4b, while both are absent in pAsa4c. However, several genes of unknown function upstream from the transfer locus were deleted from this position in pAsa4c. The two new variants also have an insertion contiguous to the *traLEKBVA* locus that is not present in pAsa4 (Fig. 1B, rainbow colored section in pAsa4b, green to purple). This region is slightly longer in pAsa4b and harbors other transfer genes and coding sequences. Interestingly, the ultimate downstream gene in this segment is a putative phage-type endonuclease that shares identity with a pAsa4 pseudogene that, given the inversion and deletion in this region, is at the same location with respect to the other coding sequences in pAsa4 (Fig. 1A, asterisk). Conjugative transfer of all pAsa4 variants in *E. coli* was attempted. JF2267 (pAsa4c) and 01-B522 (pAsa4b) were able to produce transconjugants, but A449 (pAsa4) did not.

### Comparative analysis of the pAsa4 architecture

**Figure 3:**
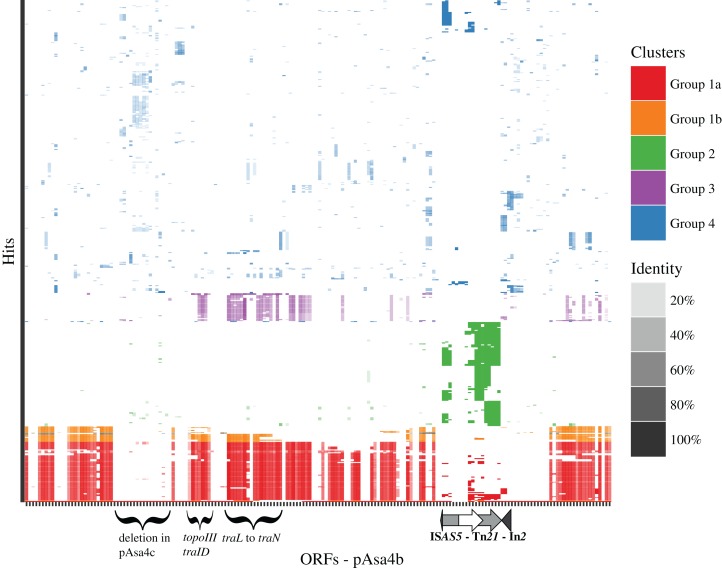
Clustering tBlastn results for pAsa4b. The shading denotes the maximum identity between the ORF query and the target. *k-means* clustered the molecules into four stable groups: Group 1 a and b is representative of incompatibility group IncA/C plasmids; Groups 2 and 4 do not encompass specific types of sequence identifiers. However, Group 2 shares significant identity with Tn*21* targets; Group 3 is representative of integrative and conjugative elements (ICEs). Some regions are less covered by tBlastn hits, such as Fig. 1B blue segment, and a region downstream from Tn*21*.

In order to find similarities between empirically drawn regions shown in Fig. 1 and co-transferred block of genes, a tBlastn search of pAsa4b coding sequences (excluding IS transposases) against the NCBI non-redundant database was achieved to collect 516 uniquely identified sequences that were hit more than three times. By *k-means* clustering, those sequences were reproducibly clustered into four groups, one of which had two sub-groups (Fig. 3). Overall, identity percentage for the hits was between 20 and 80%, except for Group 2, where the identity was near 100%. Group 1 was divided into sub-groups a and b, which would always be differentiated by the *k-means* analysis. Group 1 (Fig. 3, red and orange) had hits for coding sequences scattered along pAsa4b against the A/C_2_ family conserved backbone (Fricke et al., 2009; Harmer & Hall, 2015). pRA1, a A/C_1_ plasmid, also fell in this category (Harmer & Hall, 2015). The hits covered the majority of the plasmid, including the first and second conjugative block (Fig. 1A), but not the *tet* region (Fig. 1B, blue segment) nor the region directly downstream of IS*AS5*-Tn*21*. Group 2 (Fig. 3, green) had hits targeted at Tn*21*/In*2*. However, its sequence identifiers were more disparate. Group 3 (Fig. 3, purple) had hits against integrative conjugative elements (ICE) and the *Vibrio* STX-pathogenesis island for some of the coding sequences that provided hits in Group 1. Group 4 (Fig. 2, blue) had more heterogeneous identifiers and had hits for more specific coding sequences, including sequences for the Tn*7*-like transposition proteins and the *hipAB* toxin-antitoxin genes.

**Figure 4:**
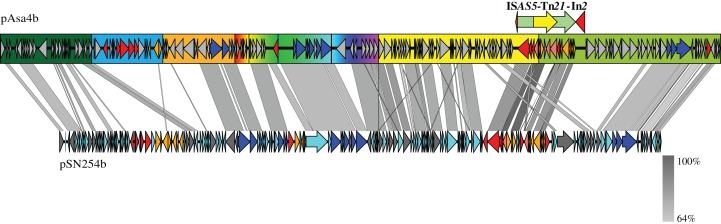
Nucleotide alignment between pAsa4b and pSN254b. Plasmid alignments and ORF representations were done with EasyFig (Sullivan, Petty & Beatson, 2011). The color codes and segments are the same as in Fig. 1.

A final alignment was performed between pAsa4b and another *A. salmonicida* subsp. *salmonicida* plasmid, pSN254b (Fig. 4). pSN254b is a large IncA/C_2_ plasmid that is also found in Canadian isolates (Vincent et al., 2014b). The identity between continuous segments was between 59 and 81%, and the synteny between genes was well conserved, a feature that could not be analyzed by the heatmap. However, due to the dissimilarity of the backbone, pAsa4s cannot be considered as an IncA/C plasmid compared to what has previously been described (Fricke et al., 2009). Again, the *tet*-containing segment (Fig. 4, blue rectangle) and a region directly downstream from Tn*21* were not covered by the alignment (Fig. 4).

### Variant genotyping and antibiotic resistances

Some insertions and deletions between pAsa4 variants changed their antibiotic resistance gene content. Consequently, A449, 01-B522 and JF2267 displayed different antibiotic resistances (Table 1). All resistance patterns but SXT, which is an antibiotic combination used in aquaculture (Morin, 2010), are directly explained by their respective pAsa4 architectures. JF2267 lack of tetracycline resistance is related to a segment deletion in pAsa4 that carries *tetA*(E) (Fig. 1B, blue segment). 01-B522 lack of chloramphenicol resistance is explained by its In*2* structure, which does not bear the IS*CR*-*cat* insertion (Fig. 2). Since pAsa4 carries those two regions, A449 is resistant to both antibiotics.

Among a collection of 129 *A. salmonicida* subsp. *salmonicida* isolates from Canada and Europe, 11 (A449 included) were detected with pAsa4-positive PCR genotyping results using a single pair of primers (Tables 1 and S2). These isolates had different resistance profiles for tetracycline, chloramphenicol and SXT resistance, and none were resistant to florfenicol, another aquaculture-relevant antibiotic whose resistance is conferred by pSN254b (Tables 1 and S1). 01-B522 was the only SXT-resistant strain, and since only the sulphonamide resistance is encoded on pAsa4, the 01-B522 genome has presumably another element to provide trimethoprim resistance. Otherwise, strains bearing pAsa4 variants could be clustered into three groups: tetracycline resistant, chloramphenicol resistant and resistant to both antibiotics.

We refined our genotyping of the pAsa4 variants by designing primers covering each segment junction (Fig. 1B; Table S2). All 11 pAsa4-positive isolates were associated with one variant version: pAsa4, pAsa4b, or pAsa4c (Table 1). All but one of the isolates (RS 1458) displayed the junction pattern (Fig. 1B) of their variant type and had a concordant antibiotic resistance profile (Table 1). The irregular strain RS 1458 had a pAsa4b pattern, except for Tn*21* (Fig. 1B, yellow to green junction). With exception of the two strains of unknown geographic origin, pAsa4b was found in Canada, while pAsa4 and pAsa4c were found in Europe (Table 1).

## Discussion

pAsa4 is a large antimicrobial resistance-encoding plasmid that was sequenced with *A. salmonicida* subsp. *salmonicida* reference genome (Reith et al., 2008). In this study, we characterized two pAsa4 variants, pAsa4b and pAsa4c. The analysis of these variants highlighted the importance of mobile genetic elements in shaping the genomic landscape of this bacterium, in particular its antibiotic resistance and its ability to propagate by conjugation. Moreover, comparative genomics between variants and other plasmids as well as base substitution analysis were used to infer pAsa4 modular architecture.

The variable position of the ISs in the pAsa4 variants indicated that they were active and capable of transposition (Fig. 1A). This is an additional example of IS activity responsible for plasmid variations in *A. salmonicida* subsp. *salmonicida* (Attéré et al., 2015; Najimi et al., 2009; Trudel et al., 2013; Vincent et al., 2014b). Moreover, pAsa4c bears IS*Kpn3*, originally described on the *Klebsiella pneumoniae* plasmid pRDDHA. Based on its transposase annotation, up to twelve copies of this IS could be found in the *Aeromonas media* WS chromosome (accession number: CP007567) and 1–3 copies could be found in *Aeromonas dhakensis* SSU (accession number: JDWD00000000.1). However, *A. media* WS may be prone to “infection” by ISs since it bears 324 transposase-associated annotations (7.4% of the coding sequence), compared to an average of 38.42 transposase genes per bacterial genomes (Aziz, Breitbart & Edwards, 2010). IS and transposase proportion varies within *Aeromonas sp.* (Chai, Wang & Chen, 2012; Vincent et al., 2016b). All these ISs can disrupt genes and functions by subsequent transposition, or can be targeted by the recombination machinery to produce larger structural variations, and thus bring a genetic modification potential. In pAsa4, two IS*AS1*s were likely the mediators of the large inversion (Fig. 1A). This is a reason why pAsa4 is not conjugative compared to pAsa4b and pAsa4c (Fig. 1B). Similar IS-dependent recombinations have been observed in pAsa5 variants in *A. salmonicida* subsp. *salmonicida* and have been reproduced *in vitro* by growing the bacteria under stressful conditions (Daher et al., 2011; Emond-Rheault et al., 2015; Tanaka et al., 2012; Vincent et al., 2016b).

In*2* is both an active mobile element in pAsa4 and a site for complex IS integration. The cassette integration system is potentially active given the presence of *aadA* cassette in pAsa4 and pAsa4c. This region also contains the *cat* gene, encoding a chloramphenicol acetyltransferase, which is not integrated as a cassette, but rather as an IS*CR*-like IS (Fig. 2). This provides another example of phenotypic diversity driven by ISs. The IS*CR* elements, which are known to transpose *cis* resistance genes between class one integrons in non-standard transpositions, may also create integron fragment duplications during those events (Toleman, Bennett & Walsh, 2006). In pAsa4, the duplication of the integrase between the IS*CR* transposase fragment and the cassette structure is an indication of a complex transposition (Fig. 2).

Empirical segments representing insertion/deletion and inversion were created to facilitate plasmid visualization and genotyping. PCR across the segments junctions paired with an antimicrobial disk assay assigned plasmid variant types to strains that bore pAsa4-like plasmids (Table 1). Some inserted/deleted blocks between variants could be related to original genes series or metabolic functions that came together by horizontal gene transfer, and provide insights for this process in *A. salmonicida*. Thus, base substitution counts (Fig. S1) and tBlastn comparisons (Fig. 3) were used to further our analysis. The heatmap and the resulting clustering revealed previously observed similarities between pAsa4 and the IncA/C incompatibility group, although pAsa4s fail the requirements to belong in either IncA/C_1_ or IncA/C_2_ (Fricke et al., 2009; Harmer & Hall, 2015). However, two pAsa4b regions were poorly covered by hits in this analysis. One was the region deleted in pAsa4c, corresponding to blue segment in Fig. 1B. Since very few hits were found against these ORFs, their origin, although not IncA/C-related, could not be inferred. However, this module could bring specific accessory functions to pAsa4-bearing strains. The other region poorly covered by hits was a region immediately downstream from Tn*21*. Interestingly, the base substitution analysis also showed that this region was not prone to mutation (Fig. S1). This region contains many genes that code for hypothetical proteins, but their implication in pAsa4 maintenance or functions is unknown. However, given their presence in all variants and the region’s low substitution rate, it could contain genes essential for pAsa4 maintenance and is another region unique to pAsa4, compared to the A/C group. The pAsa4b to pSN254b alignment further highlight those two unique regions, as well as the synteny between the common ones (Fig. 4).

## Conclusion and Perspectives

Our results showed that pAsa4 variant architecture impacts resistance antibiotic genes, and identified active ISs and integration hotspots that could promote novel resistance combinations. Because of its ubiquitous nature, *A. salmonicida* subsp. *salmonicida* interacts with many other waterborne microbes. Therefore, it may serve as a reservoir for disseminating new plasmid-based combinations of antimicrobial resistance. Even if pAsa4 was not as prevalent as pSN254b in geographic regions included in the present study, it should be regarded as a potential threat to the propagation and shuffling of antibiotic resistance due to its modular and recombinant structure. The transmission of pAsa4 should thus be monitored, especially given the propagation of *A. salmonicida* subsp. *salmonicida* infections in fish farms.

## Supplemental Information

10.7717/peerj.2595/supp-1 Supplemental Information 1 Substitution rate between pAsa4c (JF2267) and pAsa4b (01-B522) using 1-kbp windows. A global alignment of pAsa4b and pAsa4c was performed using *stretcher* (available as a part of EMBOSS 6.6.0.0) (Rice, Longden & Bleasby, 2000), and a custom R script (R Development Core Team, 2015) was used to visualize the number of substitutions by 1000-bp sliding windows (Zeileis & Grothendieck, 2005). Transparent lines represent deletions in pAsa4c compared to pAsa4b and should not be considered. Insertion track concerns small indels only.

10.7717/peerj.2595/supp-2 Supplemental Information 2 *A. salmonicida* subsp. *salmonicida* isolates used in this study.

10.7717/peerj.2595/supp-3 Supplemental Information 3 Primers used in this study to distinguish pAsa4 variants.

10.7717/peerj.2595/supp-4 Supplemental Information 4 Stretcher output and Fig. S1 code.

10.7717/peerj.2595/supp-5 Supplemental Information 5 Heatmap code and raw data for pAsa4b.

## Additional Information and Declarations

### DNA Deposition

The following information was supplied regarding the deposition of DNA sequences: GenBank pAsa4b: KT033469, pAsa4c: KT033470.

### Data Deposition

The following information was supplied regarding data availability: The raw data has been supplied as Supplemental Dataset Files.
