# Supplementary figures and images for: The mosaic architecture of Aeromonas salmonicida subsp. salmonicida pAsa4 plasmid and its consequences on antibiotic resistance
Source: PeerJ. 2016 Oct 27;4:e2595. doi: 10.7717/peerj.2595 (PMC5088629; doi:10.7717/peerj.2595)

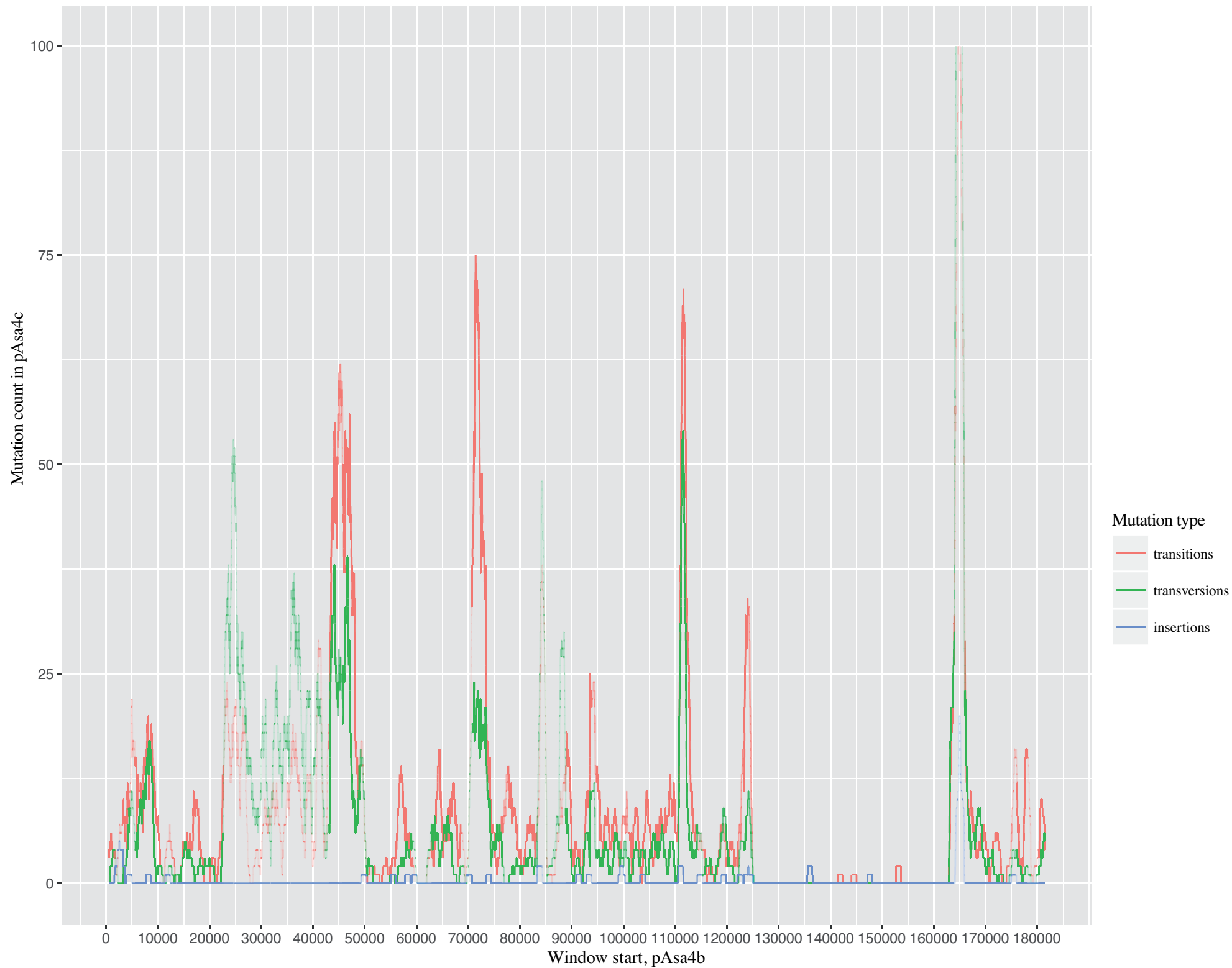

Supplement: Supplemental Information 1 — A global alignment of pAsa4b and pAsa4c was performed using stretcher (available as a part of EMBOSS 6.6.0.0) (Rice, Longden & Bleasby, 2000), and a custom R script (R Development Core Team, 2015) was used to visualize the number of substitutions by 1000-bp sliding windows (Zeileis & Grothendieck, 2005). Transparent lines represent deletions in pAsa4c compared to pAsa4b and should not be considered. Insertion track concerns small indels only. [file peerj-04-2595-s001.pdf]
