# Supplementary material for: The mosaic architecture of Aeromonas salmonicida subsp. salmonicida pAsa4 plasmid and its consequences on antibiotic resistance
Source: PeerJ. 2016 Oct 27;4:e2595. doi: 10.7717/peerj.2595 (PMC5088629; doi:10.7717/peerj.2595)
Supplement: Supplemental Information 2 [file peerj-04-2595-s002.docx]

| **Isolate** | **Source (host)*** | **Origin*** | **Antibiotic resistance determined by antibiogram†** | **pAsa4-like presence based on genotyping‡** | **Reference** |
| --- | --- | --- | --- | --- | --- |
| A449 | Brown trout | France | TET, CHL | + | ([Reith et al. 2008](#_ENREF_11)) |
| 01-B522 | Brook trout | Quebec | SXT, TET | + | ([Daher et al. 2011](#_ENREF_5)) |
| JF2267 | Arctic char | Switzerland | CHL | + | ([Braun et al. 2002](#_ENREF_2)) |
| HER1098 | INA | United States | - | - | ([Daher et al. 2011](#_ENREF_5)) |
| HER1110 | INA | Japan | - | - | ([Popoff 1971](#_ENREF_10)) |
| HER1108 | INA | Denmark | - | - | ([Popoff 1971](#_ENREF_10)) |
| HER1104 | INA | France | - | - | ([Daher et al. 2011](#_ENREF_5)) |
| HER1085 | Tout | Norway | - | - | ([Daher et al. 2011](#_ENREF_5)) |
| HER1084 | INA | France | - | - | ([Popoff 1971](#_ENREF_10)) |
| HER1107 | INA | INA | TET |  | ([Daher et al. 2011](#_ENREF_5)) |
| 01-B516 | Brook trout | Quebec | - | - | ([Daher et al. 2011](#_ENREF_5)) |
| 01-B526 | Brook trout | Quebec | - | - | ([Dautremepuits et al. 2006](#_ENREF_6)) |
| 07-9324 | Brook trout | Quebec | TET, CHL, FFC | - | ([Trudel et al. 2013](#_ENREF_13)) |
| 07-7817 | INA | Quebec | TET, CHL, FFC | - | ([Trudel et al. 2013](#_ENREF_13)) |
| 07-7346 | Atlantic salmon | Quebec | - | - | ([Trudel et al. 2013](#_ENREF_13)) |
| 07-5957 | Atlantic salmon | Quebec | - | - | ([Trudel et al. 2013](#_ENREF_13)) |
| 08-2647 | Brook trout | Quebec | SXT, TET, CHL, FFC | - | ([Trudel et al. 2013](#_ENREF_13)) |
| 09-0167 | Atlantic salmon | Quebec |  | - | ([Trudel et al. 2013](#_ENREF_13)) |
| 07-7287 | Brook trout | Quebec | TET, CHL, FFC | - | ([Trudel et al. 2013](#_ENREF_13)) |
| 08-2783 | Brook trout | Quebec | TET, CHL, FFC | - | ([Trudel et al. 2013](#_ENREF_13)) |
| 08-4188 | Brook trout | Quebec | TET, CHL, FFC | - | ([Trudel et al. 2013](#_ENREF_13)) |
| m17524-09 | Brook trout | Quebec | - | - | ([Trudel et al. 2013](#_ENREF_13)) |
| m14349-09 | Atlantic salmon | Quebec | TET, CHL, FFC | - | ([Trudel et al. 2013](#_ENREF_13)) |
| m23281-09 | Brook trout | Quebec | - | - | ([Trudel et al. 2013](#_ENREF_13)) |
| m23067-09 | Brook trout | Quebec | - | - | ([Trudel et al. 2013](#_ENREF_13)) |
| m19438-09 | Brook trout | Quebec | - | - | ([Trudel et al. 2013](#_ENREF_13)) |
| m16583-09 | Brook trout | Quebec | - | - | ([Trudel et al. 2013](#_ENREF_13)) |
| m14231-09 | Atlantic salmon | Quebec | - | - | ([Trudel et al. 2013](#_ENREF_13)) |
| m11743-09 | Brook trout | Quebec | - | - | ([Trudel et al. 2013](#_ENREF_13)) |
| m11431-09 | Brook trout | Quebec | - | - | ([Trudel et al. 2013](#_ENREF_13)) |
| m10419-09 | Brook trout | Quebec | - | - | ([Trudel et al. 2013](#_ENREF_13)) |
| m9906-09 | Brook trout | Quebec | TET, CHL, FFC | - | ([Trudel et al. 2013](#_ENREF_13)) |
| m9954-10 | Brook trout | Quebec | - | - | ([Trudel et al. 2013](#_ENREF_13)) |
| m8029-10 | Brook trout | Quebec | - | - | ([Trudel et al. 2013](#_ENREF_13)) |
| m11603-10 | Brook trout | Quebec | - | - | ([Trudel et al. 2013](#_ENREF_13)) |
| m6363-10 | Brook trout | Quebec | - | - | ([Trudel et al. 2013](#_ENREF_13)) |
| m9221-10 | Brook trout | Quebec | - | - | ([Trudel et al. 2013](#_ENREF_13)) |
| 2009-178 K9 | Atlantic salmon | New Brunswick |  | - | ([Trudel et al. 2013](#_ENREF_13)) |
| 2009-157 K5 | Brook trout | New Brunswick | TET | - | ([Trudel et al. 2013](#_ENREF_13)) |
| 2010-47 K18 | Brook trout | New Brunswick | TET, CHL, FFC | - | ([Trudel et al. 2013](#_ENREF_13)) |
| 2004-05 MF26 | INA | New Brunswick | SXT, TET, CHL, FFC | - | ([Trudel et al. 2013](#_ENREF_13)) |
| 2004-68 K52 | Atlantic salmon | Nova Scotia | SXT, TET, CHL, FFC | - | ([Trudel et al. 2013](#_ENREF_13)) |
| 2009-195 K29 | Brook trout | New Brunswick | TET | - | ([Trudel et al. 2013](#_ENREF_13)) |
| 2009-144 K3 | Brook trout | New Brunswick | SXT, TET, CHL, FFC | - | ([Trudel et al. 2013](#_ENREF_13)) |
| 2005-175 K2 | Brook trout | New Brunswick | - | - | ([Trudel et al. 2013](#_ENREF_13)) |
| M10935-11 | Brook trout | Quebec | - | - | ([Trudel et al. 2013](#_ENREF_13)) |
| M15448-11 | Brook trout | Quebec | TET, CHL, FFC | - | ([Trudel et al. 2013](#_ENREF_13)) |
| M16474-11 | Brook trout | Quebec | TET, CHL, FFC | - | ([Trudel et al. 2013](#_ENREF_13)) |
| M19878-11 | Brook trout | Quebec | SXT | - | ([Trudel et al. 2013](#_ENREF_13)) |
| M11500-11 | Brook trout | Quebec | - | - | ([Trudel et al. 2013](#_ENREF_13)) |
| M16486-11 | Brook trout | Quebec | - | - | ([Trudel et al. 2013](#_ENREF_13)) |
| M13460-11 | Brook trout | Quebec | - | - | ([Trudel et al. 2013](#_ENREF_13)) |
| M13729-11 | Brook trout | Quebec | - | - | ([Trudel et al. 2013](#_ENREF_13)) |
| M14481-11 | Brook trout | Quebec | SXT, TET, CHL, FFC | - | ([Trudel et al. 2013](#_ENREF_13)) |
| M15879-11 | Brook trout | Quebec | SXT, TET, CHL, FFC | - | ([Trudel et al. 2013](#_ENREF_13)) |
| M17739-11 | Brook trout | Quebec | SXT, TET, CHL, FFC | - | ([Trudel et al. 2013](#_ENREF_13)) |
| M13732-11 | Brook trout | Quebec | TET, CHL, FFC | - | ([Trudel et al. 2013](#_ENREF_13)) |
| M17053-11 | Brook trout | Quebec | TET, CHL, FFC | - | ([Trudel et al. 2013](#_ENREF_13)) |
| M15878-11 | Rainbow trout | Quebec | - | - | ([Trudel et al. 2013](#_ENREF_13)) |
| M13182-11 | Atlantic salmon | Quebec | - | - | ([Trudel et al. 2013](#_ENREF_13)) |
| M17735-11 | Brook trout | Quebec | - | - | ([Trudel et al. 2013](#_ENREF_13)) |
| M15576-11 | Brook trout | Quebec | - | - | ([Trudel et al. 2013](#_ENREF_13)) |
| M15469-11 | Brook trout | Quebec | TET, CHL, FFC | - | ([Trudel et al. 2013](#_ENREF_13)) |
| M22710-11 | Brook trout | Quebec | - | - | ([Trudel et al. 2013](#_ENREF_13)) |
| M13764-11 | Brook trout | Quebec | - | - | ([Trudel et al. 2013](#_ENREF_13)) |
| M18076-11 | Lumpfish | Quebec | - | - | ([Trudel et al. 2013](#_ENREF_13)) |
| M23911-11 | Brook trout | Quebec | - | - | ([Trudel et al. 2013](#_ENREF_13)) |
| RS 1458 | Rainbow trout | Ontario | TET | + | ([Attere et al. 2015](#_ENREF_1)) |
| RS 1706 | Chinook salmon | Ontario | - | - | ([Attere et al. 2015](#_ENREF_1)) |
| RS 1835 | Coho salmon | Ontario | - | - | ([Attere et al. 2015](#_ENREF_1)) |
| RS 1752 | Pumpkinseed | Ontario | - | - | ([Attere et al. 2015](#_ENREF_1)) |
| RS 1705 | Brook trout | Ontario | - | - | ([Attere et al. 2015](#_ENREF_1)) |
| RS 1744 | Coho salmon | Ontario | - | - | ([Attere et al. 2015](#_ENREF_1)) |
| RS 530 (NCMB 1102) | Atlantic salmon | Wales, United Kingdom | - | - | ([Burr & Frey 2007](#_ENREF_3); [Kupfer et al. 2006](#_ENREF_8); [Olivier et al. 1992](#_ENREF_9)) |
| RS 534 (A450) | INA | France | TET, CHL | + | ([Kay et al. 1981](#_ENREF_7)) |
| RS 887 | Coho salmon | Russia | - | - | ([Attere et al. 2015](#_ENREF_1)) |
| M12357-12 | Brook trout | Quebec | SXT, TET, CHL, FFC | - | ([Attere et al. 2015](#_ENREF_1)) |
| M21375-12 | Brook trout | Quebec | - | - | ([Attere et al. 2015](#_ENREF_1)) |
| M16237-12 | Brook trout | Quebec | - | - | ([Attere et al. 2015](#_ENREF_1)) |
| M12976-12 | Brook trout | Quebec | - | - | ([Attere et al. 2015](#_ENREF_1)) |
| M22895-12 | Brook trout | Quebec | - | - | ([Attere et al. 2015](#_ENREF_1)) |
| M10745-12 | Brown trout | Quebec | TET, CHL, FFC | - | ([Attere et al. 2015](#_ENREF_1)) |
| M9754-12 | Brook trout | Quebec | - | - | ([Attere et al. 2015](#_ENREF_1)) |
| M17930-12 | Brook trout | Quebec | - | - | ([Attere et al. 2015](#_ENREF_1)) |
| M12418-12 | Brook trout | Quebec | - | - | ([Attere et al. 2015](#_ENREF_1)) |
| M21368-12 | Brook trout | Quebec | - | - | ([Attere et al. 2015](#_ENREF_1)) |
| M13050-12 | Brook trout | Quebec | TET, CHL, FFC | - | ([Attere et al. 2015](#_ENREF_1)) |
| M24783-12 | Brook trout | Quebec | - | - | ([Attere et al. 2015](#_ENREF_1)) |
| M13566-12 | Brook trout | Quebec | SXT, TET, CHL, FFC | - | ([Attere et al. 2015](#_ENREF_1)) |
| M14404-12 | Atlantic salmon | Quebec | - | - | ([Attere et al. 2015](#_ENREF_1)) |
| M16671-12 | Brook trout | Quebec | - | - | ([Attere et al. 2015](#_ENREF_1)) |
| M16042-12 | Brook trout | Quebec | SXT, TET, CHL, FFC | - | ([Attere et al. 2015](#_ENREF_1)) |
| SHY13-162 | Brook trout | Quebec | - | - | ([Attere et al. 2015](#_ENREF_1)) |
| SHY13-574 | Brook trout | Quebec | - | - | ([Attere et al. 2015](#_ENREF_1)) |
| SHY13-1470 | Brook trout | Quebec | - | - | ([Attere et al. 2015](#_ENREF_1)) |
| SHY13-2188 | Brook trout | Quebec | SXT, TET, CHL, FFC | - | ([Attere et al. 2015](#_ENREF_1)) |
| SHY13-2222 | Brook trout | Quebec | SXT | - | ([Attere et al. 2015](#_ENREF_1)) |
| SHY13-2257 | Brook trout | Quebec | - | - | ([Attere et al. 2015](#_ENREF_1)) |
| SHY13-2263 | Brook trout | Quebec | - | - | ([Attere et al. 2015](#_ENREF_1)) |
| SHY13-2317 | Brook trout | Quebec | - | - | ([Attere et al. 2015](#_ENREF_1)) |
| SHY13-2425 | Brook trout | Quebec | - | - | ([Attere et al. 2015](#_ENREF_1)) |
| SHY13-2458 | Brook trout | Quebec | SXT, TET, CHL, FFC | - | ([Attere et al. 2015](#_ENREF_1)) |
| SHY13-2534 | Atlantic salmon | Quebec | - | - | ([Attere et al. 2015](#_ENREF_1)) |
| SHY13-2627 | Brook trout | Quebec | TET | + | ([Attere et al. 2015](#_ENREF_1)) |
| SHY13-2630 | Brook trout | Quebec | SXT, TET, CHL, FFC | - | ([Attere et al. 2015](#_ENREF_1)) |
| SHY13-2825 | Brook trout | Quebec | - | - | ([Attere et al. 2015](#_ENREF_1)) |
| SHY13-2873 | Brook trout | Quebec | - | - | ([Attere et al. 2015](#_ENREF_1)) |
| SHY13-2909 | Brook trout | Quebec | - | - | ([Attere et al. 2015](#_ENREF_1)) |
| SHY13-3101 | Brook trout | Quebec | - | - | ([Attere et al. 2015](#_ENREF_1)) |
| SHY13-3127 | Brook trout | Quebec | - | - | ([Attere et al. 2015](#_ENREF_1)) |
| SHY13-3795 | Brook trout | Quebec | TET, CHL, FFC | - | ([Attere et al. 2015](#_ENREF_1)) |
| SHY13-3798 | Brook trout | Quebec | - | - | ([Attere et al. 2015](#_ENREF_1)) |
| SHY13-3799 | Brook trout | Quebec | TET | + | ([Attere et al. 2015](#_ENREF_1)) |
| JF2506 | Atlantic salmon | Norway | - | - | ([Burr & Frey 2007](#_ENREF_3); [Olivier et al. 1992](#_ENREF_9)) |
| JF2507 | Atlantic salmon | Scotland, United Kingdom | - | - | ([Burr & Frey 2007](#_ENREF_3); [Olivier et al. 1992](#_ENREF_9)) |
| JF2510 | Atlantic salmon | Norway | - | - | ([Burr & Frey 2007](#_ENREF_3); [Olivier et al. 1992](#_ENREF_9)) |
| JF3224 | Brown trout | Switzerland | - | - | ([Burr & Frey 2007](#_ENREF_3); [Burr et al. 2005](#_ENREF_4)) |
| JF3327 | Arctic char | Switzerland | - | - | ([Burr & Frey 2007](#_ENREF_3)) |
| JF3496 | Brown trout | Switzerland | - | - | ([Burr & Frey 2007](#_ENREF_3)) |
| JF3517 | Turbot | Norway | CHL | + | ([Burr & Frey 2007](#_ENREF_3)) |
| JF3518 | Turbot | Norway | CHL | + | ([Burr & Frey 2007](#_ENREF_3)) |
| JF3844 | Arctic char | Switzerland | - | - | ([Burr & Frey 2007](#_ENREF_3)) |
| JF2869 | Arctic char | INA | CHL | + | ([Studer et al. 2013](#_ENREF_12)) |
| JF3223 | Brown trout | Switzerland | - | - | ([Burr & Frey 2007](#_ENREF_3)) |
| JF3519 | Arctic char | Switzerland | - | - | ([Burr & Frey 2007](#_ENREF_3)) |
| JF3791 | Arctic char | Switzerland | CHL | - | ([Burr & Frey 2007](#_ENREF_3)) |
| JF4111 | Arctic Char | Switzerland | - | - | ([Studer et al. 2013](#_ENREF_12)) |
| JF4112 | Arctic Char | Switzerland | - | - | ([Studer et al. 2013](#_ENREF_12)) |
| JF4113 | Arctic Char | Switzerland | - | - | ([Studer et al. 2013](#_ENREF_12)) |

* INA = information not available.

† Resistances to SXT = sulfamethoxazole/trimethoprim (23.75 µg/1.25 µg), TET = tetracycline (5 µg), FCC = florfénicol (30 µg) and CHL = chloramphenicol (30 µg) (Becton Dickinson, USA) were assayed as previously described ([Trudel et al. 2013](#_ENREF_13)).

‡ Genotyping was done using primers named in Table S2.

**References for this table**

Attere SA, Vincent AT, Trudel MV, Chanut R, and Charette SJ. 2015. Diversity and Homogeneity among Small Plasmids of *Aeromonas salmonicida* subsp. *salmonicida* Linked with Geographical Origin. *Front Microbiol* 6:1274. 10.3389/fmicb.2015.01274

Braun M, Stuber K, Schlatter Y, Wahli T, Kuhnert P, and Frey J. 2002. Characterization of an ADP-ribosyltransferase toxin (AexT) from *Aeromonas salmonicida* subsp *salmonicida*. *Journal of Bacteriology* 184:1851-1858. 10.1128/jb.184.7.1851-1858.2002

Burr SE, and Frey J. 2007. Analysis of type III effector genes in typical and atypical *Aeromonas salmonicida*. *Journal of Fish Diseases* 30:711-714. 10.1111/j.1365-2761.2007.00859.x

Burr SE, Pugovkin D, Wahli T, Segner H, and Frey J. 2005. Attenuated virulence of an *Aeromonas salmonicida* subsp. *salmonicida* type III secretion mutant in a rainbow trout model. *Microbiology* 151:2111-2118. 151/6/2111 [pii]10.1099/mic.0.27926-0

Daher RK, Filion G, Tan SG, Dallaire-Dufresne S, Paquet VE, and Charette SJ. 2011. Alteration of virulence factors and rearrangement of pAsa5 plasmid caused by the growth of *Aeromonas salmonicida* in stressful conditions. *Veterinary Microbiology* 152:353-360. S0378-1135(11)00264-1 [pii]10.1016/j.vetmic.2011.04.034

Dautremepuits C, Fortier M, Croisetiere S, Belhumeur P, and Fournier M. 2006. Modulation of juvenile brook trout (*Salvelinus fontinalis*) cellular immune system after *Aeromonas salmonicida* challenge. *Veterinary Immunology and Immunopathology* 110:27-36. 10.1016/j.vetimm.2005.09.008

Kay WW, Buckley JT, Ishiguro EE, Phipps BM, Monette JPL, and Trust TJ. 1981. Purification and disposition of a surface protein associated with virulence of *Aeromonas salmonicida*. *Journal of Bacteriology* 147:1077-1084.

Kupfer M, Kuhnert P, Korczak BM, Peduzzi R, and Demarta A. 2006. Genetic relationships of *Aeromonas* strains inferred from 16S rRNA, *gyrB* and *rpoB* gene sequences. *Int J Syst Evol Microbiol* 56:2743-2751. 10.1099/ijs.0.63650-0

Olivier G, Moore AR, and Fildes J. 1992. Toxicity of *Aeromonas salmonicida* cells to Atlantic salmon *Salmo salar* peritoneal macrophages. *Developmental and Comparative Immunology* 16:49-61.

Popoff M. 1971. Etude sur les *Aeromonas salmonicida*. II. Caractérisation des bactériophages actifs sur les *Aeromonas salmonicid*a et lysotypie. *Annales de Recherches Veterinaires* 2:33-45.

Reith ME, Singh RK, Curtis B, Boyd JM, Bouevitch A, Kimball J, Munholland J, Murphy C, Sarty D, Williams J, Nash JH, Johnson SC, and Brown LL. 2008. The genome of *Aeromonas salmonicida* subsp. *salmonicida* A449: insights into the evolution of a fish pathogen. *BMC Genomics* 9:427. 10.1186/1471-2164-9-427

Studer N, Frey J, and Vanden Bergh P. 2013. Clustering subspecies of *Aeromonas salmonicida* using IS*630* typing. *BMC Microbiol* 13:36. 10.1186/1471-2180-13-36

Trudel MV, Tanaka KH, Filion G, Daher RK, Frenette M, and Charette SJ. 2013. Insertion sequence *AS5* (IS*AS5*) is involved in the genomic plasticity of *Aeromonas salmonicida*. *Mob Genet Elements* 3:e25640. 10.4161/mge.25640
