# Supplementary material for: The mosaic architecture of Aeromonas salmonicida subsp. salmonicida pAsa4 plasmid and its consequences on antibiotic resistance
Source: PeerJ. 2016 Oct 27;4:e2595. doi: 10.7717/peerj.2595 (PMC5088629; doi:10.7717/peerj.2595)
Supplement: Supplemental Information 3 [file peerj-04-2595-s003.docx]

| Primer | Sequence (5' to 3') | Color code (Fig. 1(b)) | Belongs to pair | Reference |
| --- | --- | --- | --- | --- |
| Deletion in pAsa4c | | | | |
| pAsa4-LTG20_21-F1 | GCCTACCGAAGACATAGCTAAAGC |  | 1, 3 | This study |
| pAsa4-LTG20_21-R1 | GTACCCAGATTATCCTTTGGCACC |  | 1 | This study |
| pAsa4-LTG3_4-F1 | ACAGGAGGCATTTAATGTTCAGAC |  | 2 | This study |
| pAsa4-LTG3_4-R1 | AGCTGATGAAGGACTGAATCTGTC |  | 2, 3 | This study |
| Rainbow block comprising large conjugative block | | | | |
| pAsa4-LTG165-F1 | ACTTATAAGACCATCCTGACGGC |  | 4, 5, 6 | This study |
| pAsa4-LTG163-R1 | AAGATCTTCACCTTGTTTATTGCCC |  | 4 | This study |
| pAsa4-traL-R1 | GCCCAATAGATCATGTGGAGTAGG |  | 5 | This study |
| pAsa4-LTG152-F1 | TGAGATATCTTTCCAGTCCACACC |  | 6, 7 | This study |
| pAsa4-LTJ014-F1 | GTGGATTGAGTGTGTGATTTACCG |  | 8 | This study |
| pAsa4-LTJ015-R1 | ATCATAAAGACGGTACTCCTCAGC |  | 7 | This study |
| pAsa4-LTJ015-R2 | AAGTAGAAGACTAACCAGCCTTGC |  | 8 | This study |
| Integron | | | | |
| pAsa4-intI-F1 | AAGCTCTCGGGTAACATCAAGG |  | 9, 10 | This study |
| pAas4-cat-R3 | CAAACATGAAAGCCATCACAAACG |  | 9 | This study |
| pAsa4-ISCR-F2 | GGAAACTCAGCACCCATTGCC |  | 11 | This study |
| pAsa4-qacE-R1 | TTTGTGTAGGGCTTATTATGCACG |  | 10, 11 | This study |
| Positive chromosomal control | | | | |
| *tapA* F | ACATGAAGAAGCAATCAGGC | NA | 12 | ([Ebanks et al. 2006](#_ENREF_1)) |
| *tapA* R | AGAGGTCATGCGTTAGCAG | NA | 12 | ([Ebanks et al. 2006](#_ENREF_1)) |
| General detection of pAsa4 and preliminary genotyping | | | | |
| DD1-pAsa4-traG-F1 | AGGTTGCTCTGGAAAGCCTCTGAT | NA | 13 | ([Vincent et al. 2014](#_ENREF_2)) |
| DD2-pAsa4-traG-R1 | TGTGGATGCCTGTGCTCTCCATTA | NA | 13 | ([Vincent et al. 2014](#_ENREF_2)) |

References for this table

Ebanks RO, Knickle LC, Goguen M, Boyd JM, Pinto DM, Reith M, and Ross NW. 2006. Expression of and secretion through the *Aeromonas salmonicida* type III secretion system. *Microbiology* 152:1275-1286. 152/5/1275 [pii]10.1099/mic.0.28485-0

Vincent AT, Trudel MV, Paquet VE, Boyle B, Tanaka KH, Dallaire-Dufresne S, Daher RK, Frenette M, Derome N, and Charette SJ. 2014. Detection of variants of the pRAS3, pAB5S9, and pSN254 plasmids in *Aeromonas salmonicida* subsp. *salmonicida*: multidrug resistance, interspecies exchanges, and plasmid reshaping. *Antimicrobial Agents and Chemotherapy* 58:7367-7374. 10.1128/AAC.03730-14
